# Diversity of *Salmonella* serotypes from humans, food, domestic animals and wildlife in New South Wales, Australia

**DOI:** 10.1186/s12879-018-3563-1

**Published:** 2018-12-05

**Authors:** Kelly M. J. Simpson, Grant A. Hill-Cawthorne, Michael P. Ward, Siobhan M. Mor

**Affiliations:** 1 School of Veterinary Science, Faculty of Science, University of Sydney, Camperdown, New South Wales Australia; 2 School of Public Health, University of Sydney, Camperdown, New South Wales Australia; 3 Marie Bashir Institute for Infectious Disease and Biosecurity, University of Sydney, Westmead, New South Wales Australia; 4 Institute of Infection and Global Health, University of Liverpool, Merseyside, Liverpool UK

**Keywords:** *Salmonella*, One Health, Wildlife, Companion animals, Livestock, Food, Serotype

## Abstract

**Background:**

*Salmonella* is an important human pathogen in Australia and annual case rates continue to increase. In addition to foodborne exposures, cases have been associated with animal and contaminated environment contact. However, routine surveillance in Australia has tended to focus on humans and food, with no reported attempts to collate and compare *Salmonella* data from a wider range of potential sources of exposure.

**Methods:**

*Salmonella* data from humans, food, animals and environments were collated from a range of surveillance and diagnostic sources in New South Wales (NSW). Data were categorised to reflect one of 29 sample origins. Serotype diversity was described for each category, and the distribution of serotypes commonly isolated from humans was examined for each sample origin. The distribution of serotypes along the livestock-food-human continuum and at the companion animal-wildlife interface was also examined.

**Results:**

In total, 49,872 *Salmonella* isolates were included in this analysis, comprising 325 serotypes. The vast majority of these isolates were from humans (*n* = 38,106). Overall *S*. Typhimurium was the most frequently isolated serotype and was isolated from all sample categories except natural environment and game meat. *S.* Enteriditis was not isolated from any livestock animal, however sporadic cases were documented in food, companion animals and a reptile. Many serotypes that were frequently isolated from livestock animals and associated food products were only rarely isolated from humans. In addition, a number of key human serotypes were only sporadically isolated from livestock and food products, suggesting alternative sources of infection. In particular, *S.* Paratyphi B Java and *S.* Wangata were more often isolated from wild animals*.* Finally, there was some overlap between serotypes in companion animals and wildlife, with cats in particular having a large number of serotypes in common with wild birds.

**Conclusions:**

This is the most comprehensive description of *Salmonella* data from humans, food, livestock, wildlife, companion animals and various environments in Australia reported to date. Results confirm that livestock and food are important sources of salmonellosis in humans but that alternative sources - such as contact with wildlife and environments - warrant further investigation. Surveillance in NSW is largely human-focussed: major knowledge gaps exist regarding the diversity and frequency of serotypes in animals. More systematic surveillance of domestic animals and wildlife is needed to inform targeted control strategies and quantitative source attribution modelling in this state.

**Electronic supplementary material:**

The online version of this article (10.1186/s12879-018-3563-1) contains supplementary material, which is available to authorized users.

## Background

Foodborne gastroenteritis in Australia is estimated to cost ~ $811 million annually due to cost of treatment, morbidity, business productivity, and government surveillance and investigation [1]. *Salmonella* is the second leading cause of gastroenteritis in the country [2] and is the most common cause of death from foodborne-related diseases worldwide [3]. Incidence of salmonellosis continues to rise in Australia each year despite notable reductions in incidence in other developed countries [4–6]. In 2014, the rate of salmonellosis cases in Australia (69.3 cases per 100,000) [7] was more than four times the rate of cases in the United States (15.45 cases per 100,000) [4]. Notably, Australia is considered free from *S*. Enteritidis in poultry [8, 9], which is the most common serotype reported in the US [4].

New South Wales (NSW) is the most populous state in Australia and accounts for approximately a quarter of the annual notified cases of *Salmonella* in humans nationally [2]. While foodborne transmission is predominant, a number of outbreaks have been associated with serotypes that are unique to NSW and that are believed (or confirmed) to be from an environmental or wildlife source [10, 11]. All human cases of salmonellosis in NSW are serotyped and reported to the state health department. Isolates may also undergo additional typing using multi-locus variable number tandem repeats assay (MLVA). Increasingly, whole genome sequencing is also being applied to determine relatedness of isolates [12, 13]. In contrast, notification of *Salmonella* cases in livestock in NSW is only mandatory for five serotypes (*S*. Gallinarum, *S*. Pullorum, *S*. Abortusequi, *S.* Abortusovis, and *S*. Enteriditis if isolated from poultry). Serotyping is not routinely performed during livestock investigations and thus many cases go unreported. Furthermore, cases diagnosed in companion animals and wildlife are not required to be reported to government agencies. This means that surveillance data on *Salmonella* infection in animals in NSW, and indeed much of Australia, are vastly lacking. Moreover, data often remain in local repositories and, even when data are collated, there are barriers to comparison because of missing metadata.

Source attribution modelling is an important tool in identifying and prioritising sources of *Salmonella* infection in humans [14]. Nationwide attribution studies have proved challenging in Australia due to state-to-state differences in surveillance and laboratory methods for *Salmonella* detection [15]. Previous comparative studies have therefore been limited to investigation of salmonellosis in one state, namely South Australia, and this study was limited to livestock and livestock products only [15]. Given the importance of non-food serotypes in NSW, we undertook an exploratory analysis of data available on *Salmonella* serotypes in this state with the aim of describing the diversity of serotypes in humans as well as food products, domestic animals and wildlife, with a view to informing future source attribution studies.

## Methods

### Data abstraction

*Salmonella* data were collated from various human and animal surveillance/diagnostic institutes: the Notifiable Conditions Incident Management System (NCIMS), NSW Food Authority (NSWFA), the Australian Registry of Wildlife Health (ARWH), electronic Wildlife Health Information System (eWHIS), the NSW State Veterinary Diagnostic Laboratory (SVDL), National Enteric Pathogen Surveillance System (NEPSS), and a major private veterinary lab, IDEXX Laboratories Pty Ltd. A brief description of each organisation/system is provided in Additional file 1. Given overlap between NCIMS and NEPSS, only non-human data were requested from the latter. For all other data sets, all available *Salmonella*-positive results were extracted. Data were excluded if the location was outside of NSW, or if serotype, date or sample origin were missing.

Since an individual isolate may have been included in multiple data sets, the combined data set was screened for duplicates. Isolates were considered “duplicated” if the sample origin, serotype and month/year were identical. Where there was an ambiguous term for sample origin, e.g. “meat”, with no further information provided, the isolate was considered a duplicate if a meat case of any type in another data set met the other requirements of a duplicate. Where a duplicate was suspected, e.g. identical sample origin and serotype but differed by one month, a conservative approach was taken and the isolates were considered non-duplicates.

### Serotype diversity and distribution

**Fig. 1:**
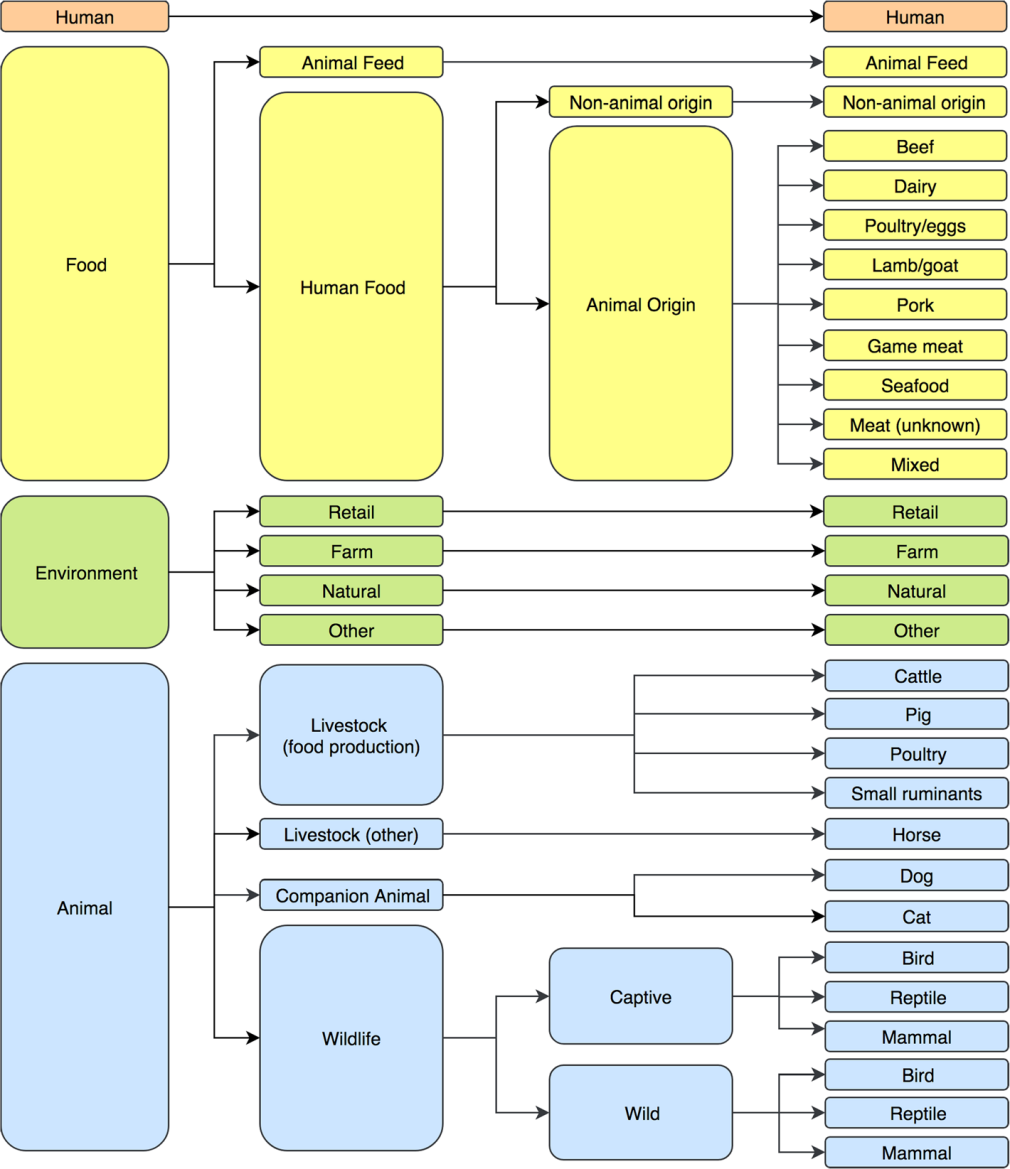
Flow chart detailing the categorisation of *Salmonella* isolates from New South Wales, Australia, based on origin of sample (*n* = 29 categories). Note that poultry includes both broilers and layers

Isolates were allocated into 29 categories based on the origin of the sample (Fig. 1). Lists of the detailed sample origins are provided in Additional file 2. For each category, frequency tables were used to identify the 10 most common serotypes. This is in accordance with public health reports and other studies in which the most frequent five or ten serotypes are typically reported [6, 16–18]. The diversity of serotypes within each category was described using Simpson’s index of diversity. Simpson’s index of diversity is commonly used in ecology to identify differences in species diversity between locations [19, 20]. It has also been used to compare the diversity of *Salmonella* populations [21, 22]. The index is a value scaled between 0 and 1; higher values represent greater diversity. The distribution of serotypes commonly isolated from humans was examined for each sample origin. In addition, we examined the distribution of serotypes along the livestock-food-human continuum and at the companion animal-wildlife interface.

## Results

Details of the data used in this study are shown in Table 1. After screening, 863 isolates were excluded from NEPSS and 1 isolate was excluded from eWHIS as they met the criteria for a duplicate in another data set. Non-serotyped positive results were excluded from NCIMS (*n* = 2201), SVDL (*n* = 123), ARWH (*n* = 12) and NEPSS (*n* = 1). The remaining 49,872 *Salmonella* isolates were included in this analysis, comprising 325 serotypes. The resolution of the molecular data differed for each data source. NCIMS and NSWFA contained more detailed molecular level data, reporting the MLVA type in 38.2 and 12.5% of isolates, respectively. Overall, human data had the most extensive representation with the largest number of samples and the most detailed molecular data (MLVA) available. In contrast, data from animal samples were limited to phage typing, which was only rarely performed. Given the lack of comparability of data at higher levels of molecular resolution, further comparisons between categories were limited to evaluation of serotypes.

**Table 1:** Sources of data on *Salmonella* used in this study

Name data source	Acronym	Dates included	Samples included	Reason for sampling	Molecular data	No. isolates w. MLVA/Phage (%)	No. isolates
Notifiable Conditions Incident Management System, NSW Health	NCIMS	2001–2015	Human	Diagnostic	MLVA and Phage	MLVA: 14550 (38.2) Phage: 14996 (39.4)	38,106
New South Wales Food Authority, Department of Primary Industries	NSWFA	2010–2017	Food, Environment	Surveillance, Outbreak- investigation	MLVA	126 (12.5)	1006
State Veterinary Diagnostic Laboratory, Department of Primary Industries	SVDL	2006–2015	Livestock, Wildlife (captive [zoo] and free-ranging)	Diagnostic, Research, Surveillance	Phage	25 (4.4)	569
Australian Registry Wildlife Health, Taronga Zoo	ARWH	1989–2015	Wildlife (captive [zoo] and free-ranging)	Diagnostic	Phage	103 (21.7)	475
Electronic Wildlife Health Information System, Wildlife Health Australia	eWHIS	2012–2016	Wildlife (free-ranging)	Diagnostic, Research, Surveillance	Phage	2 (40.0)	5
IDEXX Laboratories Pty Ltd	IDEXX	2012–2017	Dog and cat	Diagnostic	None		407
National Enteric Pathogen Surveillance System, Microbiology Diagnostic Unit	NEPSS	2006–2017	Non-human	All	None		9304
						Total isolates	49,872

Where national data were available, only data from NSW are described

**Fig. 2:**
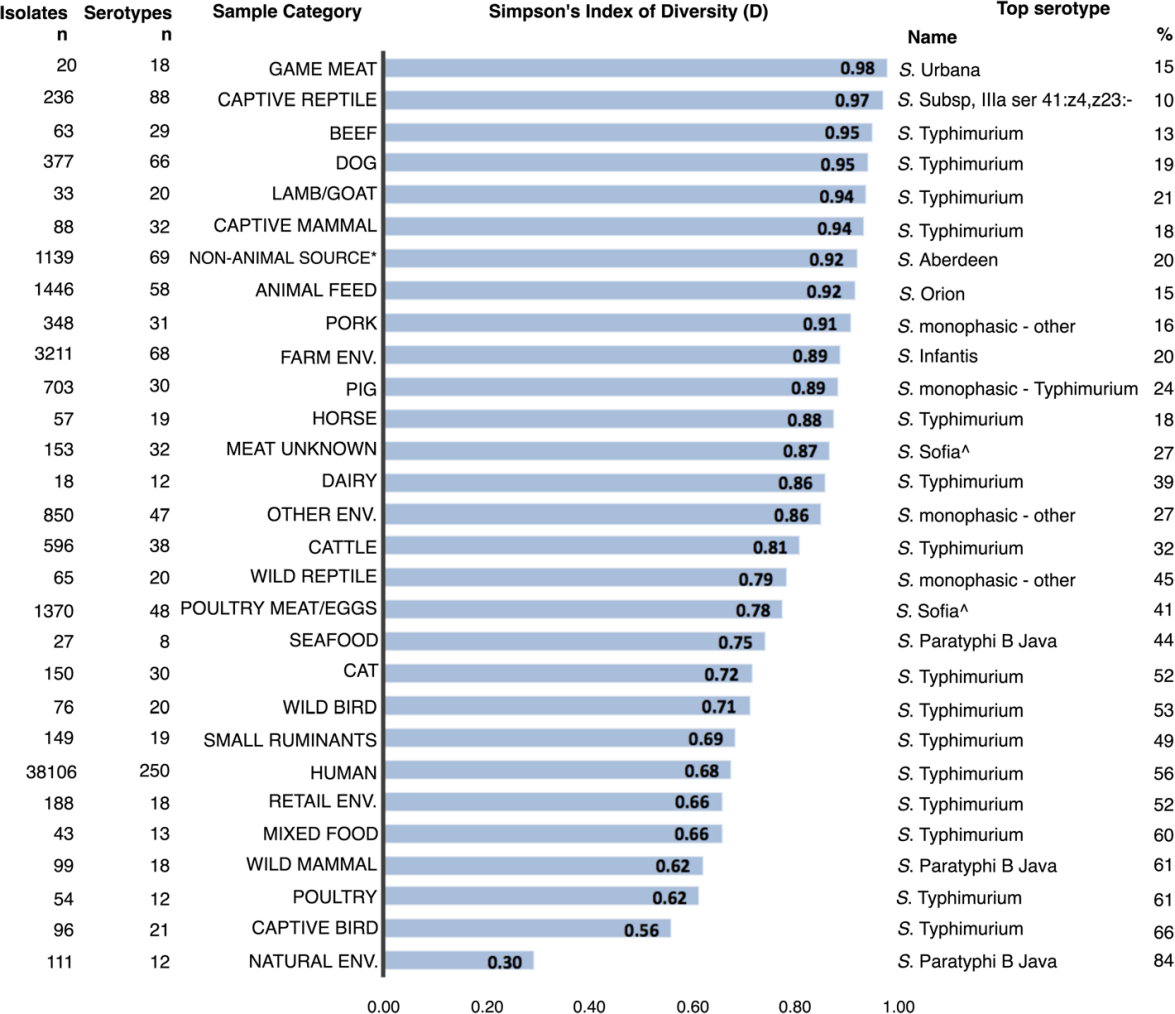
The Simpson’s index of diversity (*D*) for each sample category. Sample categories are ranked in order of most diverse to least diverse, with higher values of *D* reflecting a greater diversity of serotypes within that sample category. The most frequently isolated serotype from each sample category and percentage of isolates within the sample category comprising the top serotype are also given. Monophasic-other refers to serotypes, other than *S*. Typhimurium, that are missing an H antigen and are therefore not able to be typed as a particular serotype. *Refers to non-animal sourced foods. ^ Subsp II ser 1,4,12,27:b:[e,n,x] (Sofia)

Figure 2 shows, for each sample category, the number of isolates and serotypes, Simpson’s index of diversity (*D*) and the most frequent serotype isolated. A full listing of the ten most frequently isolated serotypes in each category is provided in Additional file 3. *D* ranged from 0.30 (natural environment; low diversity) to 0.98 (game meat; high diversity). Overall the median *D* was 0.86, illustrating that most sample categories had a high level of serotype diversity. *S*. Typhimurium was the top ranked serotype for the majority (15 of 29) of sample categories.

**Fig. 3:**
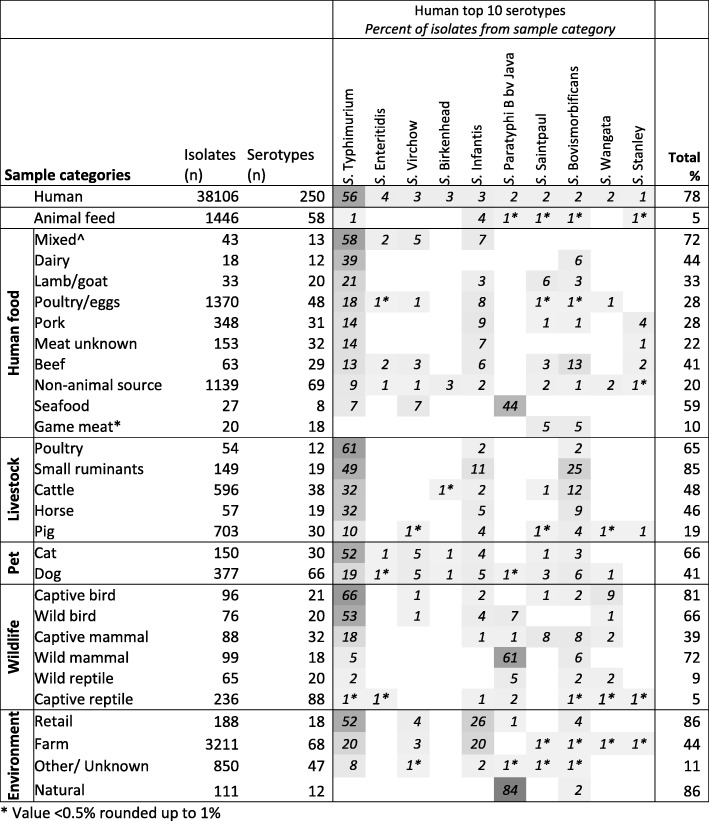
Top ten most frequently isolated serotypes in humans and their occurrence in each of the other sample categories. The numbers of samples and serotypes from each sample category are indicated on the left. The heat map gives the percentage of samples from each sample category that shared a serotype with one of the ten most frequently isolated serotypes in humans. Shade is proportional percentage ranging from light grey (low percentage of samples attributable to that serotype) to dark grey (high percentage of samples attributable to that serotype). *Game meat refers to the meat from wild caught kangaroos (*n* = 18), wild boar (*n* = 1), and a crocodile (*n* = 1). ^ Mixed refers to food that is made up of multiple food types, for example a hamburger could be a mix of beef (patty), dairy (cheese) and eggs (mayonnaise)

The proportion of the ten most frequently isolated serotypes detected in humans across each sample category is shown in Fig. 3. As expected, serotypes were not distributed evenly between the sample categories and no category was associated with all top 10 human serotypes. *S*. Typhimurium, *S*. Infantis, and *S*. Bovismorbificans were common to the majority of sample categories (27, 23 and 25 of 29 categories, respectively); overall, *S*. Typhimurium predominated. There were only two categories (natural environment and game meat) from which *S*. Typhimurium was not isolated. *S*. Enteritidis was not isolated from any livestock animal (including broilers and layers), however it was sporadically isolated in food, companion animals and a reptile. *S*. Paratyphi B Java was predominately associated with three sample categories, namely seafood, natural environment and wild mammals. *S*. Wangata, to a lesser degree, showed a higher frequency of isolation in wildlife species.

**Fig. 4:**
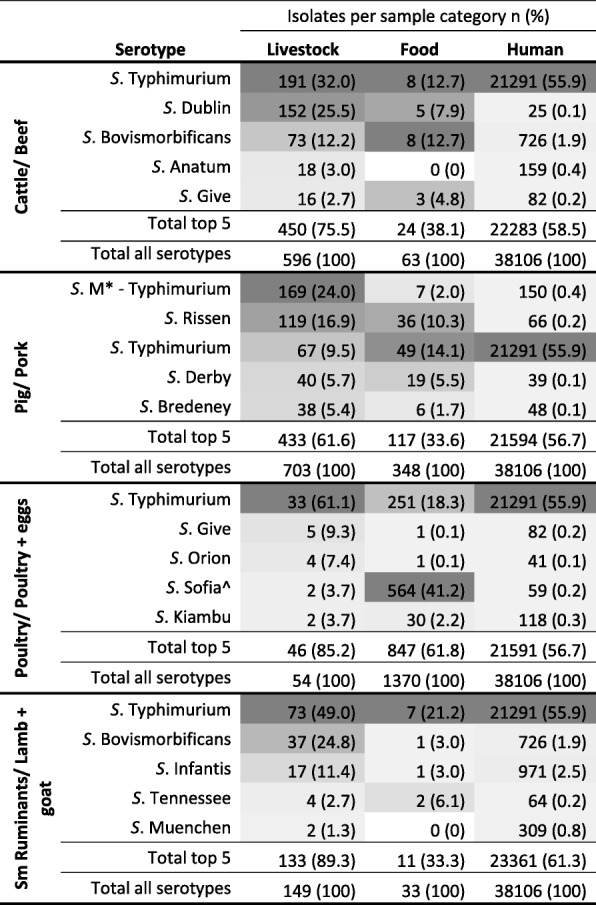
Five most frequently isolated serotypes of the four major livestock categories and the number and proportion of these serotypes in associated food commodities and humans. Shading is proportional to all the isolates from each sample type. E.g. Shading of cattle serotypes is proportional to the rest of the cattle serotype proportions. Darker shows a higher proportion of isolates attributable to that serotype from that category. *M = monophasic ^ Subsp II ser 1,4,12,27:b:[e,n,x] (Sofia)

Figure 4 shows the overlap between the five most frequent serotypes in each livestock species, their associated food commodities and humans. The only serotype that was frequently observed across livestock, associated food commodities and humans was *S*. Typhimurium. Other serotypes, such as *S.* Bovismobificans and *S*. Rissen, were observed frequently in livestock and associated food commodities but were infrequently observed in humans. In contrast, one serovar (*S*. Sofia) was commonly isolated from food products derived from poultry, but comparatively infrequently isolated from poultry and humans.

Table 2 shows the overlap between serotypes in companion animals and wildlife. There were 268 and 133 isolates from dogs and cats, respectively, that were of a serotype that was also isolated from wildlife. The highest similarity was seen between wild birds and cats, with 81% of isolates in cats being of a serotype that was also isolated from wild birds.

**Table 2:** Number of serotypes (n) in common between companion animals and wild birds, wild reptiles and wild mammals

	Dog		Cat	
	n	%	n	%
Wild birds	20	52	11	81
Wild reptiles	16	42	8	71
Wild mammals	15	38	7	63

The proportion (%) of isolates in dogs and cats that were associated with a serotype that was also isolated in wildlife is also shown

## Discussion

Australia has one of the highest incidences of human salmonellosis of any developed country [4–6, 23], yet the distribution of serotypes among different hosts is poorly understood and documented. This study provides the most comprehensive description of the distribution of *Salmonella* serotypes in NSW (and indeed Australia) to date, including humans, food products, animals (both domestic and wild) and the environment. When followed up with comprehensive prevalence surveys in animals, these results provide essential information for future source attribution studies.

Data on *Salmonella* in NSW are heavily skewed towards humans; more than three-quarters of isolates included in the study were from humans. Furthermore, detailed typing (such as MLVA) was lacking in most other data sources, with the exception of food, being an obvious delineation of public health surveillance. *S*. Typhimurium is prioritised by public health departments for molecular analysis, including whole genome sequencing [12], due to the need to monitor for emergence of new strains and to distinguish between outbreak isolates [24]. The use of these technologies to distinguish related cases is supported in this study by the relatively low diversity index of serotypes isolated from human samples (*D* = 0.68) likely due to the large proportion of *S*. Typhimurium isolates. However, given the frequency of *S*. Typhimurium in most sample categories, restricting this technology to human isolates limits the capacity for detailed source attribution.

Data presented here support the established importance of livestock and food as sources of salmonellosis. Three of the ten most frequent human serotypes (*S*. Typhimurium, *S.* Infantis, *S.* Bovismorbificans) accounted for more than 60% of human isolates, and were isolated from farm, livestock, livestock-associated food commodities, and retail samples. In particular *S*. Typhimurium - a serotype commonly associated with foodborne outbreaks - was found in all livestock species (ranging from 10 to 61% of isolates from livestock; Fig. 3). Given the documented wide host range of *S*. Typhimurium [25–27], it was unsurprising that it was a common serotype in all livestock and all associated food commodities. Nevertheless, eggs remain the most commonly implicated food in outbreaks of this serotype [2, 15]. This is likely due, in part, to unsafe food practises associated with eggs - for example their raw consumption in ready-to-eat products such as mayonnaise and tiramisu [28]. Evidence of the potential for cross-contamination at the retail level can be seen in this study, with the high percentage (86%) of isolates from the retail environment sharing a serotype with the 10 most frequently isolated human serotypes (Fig. 3). Interestingly, the diversity indices for all livestock-associated food commodities were higher than the associated livestock. Speculatively, this may be indicative of exposure to additional sources during processing (e.g. cross-contamination); this requires further research to confirm.

Not all *Salmonella* serotypes frequently found on farms, in livestock and in food are also frequently isolated from humans. Figure 4 illustrates a number of serotypes that were frequently isolated in both livestock and their associated food commodity but were rarely isolated in humans. Some serotypes are known to be host-adapted, meaning they will cause disease/infect one particular species more than others [29]. For example *S*. Dublin - which is host-adapted to cattle [29] - was isolated from more than a quarter of cattle samples but less than 0.1% of human samples (Fig. 4). As such, surveillance methods that do not serotype *Salmonella* isolates or use culture independent laboratory techniques, e.g. PCR testing of meat juice or seroprevalence surveys on-farm, may not be the most appropriate method for determining the safety of food or contribution of livestock to human illness.

The overall similarity between the types and frequencies of serotypes isolated in humans and companion animals is consistent with other studies that hypothesise companion animals might serve as reservoirs for salmonellosis [30, 31]. The source of *Salmonella* infection in companion animals is often due to pet food [32, 33] but this is not exclusive. Hunting behaviours of cats has also been linked to *Salmonella* transmission from wild birds [34–36], supported by results from this study: 81% of cat isolates shared a common serotype with wild birds (Table 2). This suggests that in addition to being a reservoir, companion animals might also act as vectors of transmission between the environment/wildlife and their owners. The idea of companion animals as reservoirs or vectors for *Salmonella* is of concern for human health for two reasons. Firstly, companion animals are frequently treated with antimicrobials so there is the potential to influence antimicrobial resistance patterns. An illustration of this can be seen in a recent case study in Sydney that described carbapenemase-producing *Salmonella* infections in cats [37]. Secondly, companion animals often have an intimate relationship with their owners that involves frequent direct touching (e.g. licking) and use of a shared environment, which increases the risk of transmission. Although prevalence of *Salmonella* in cats and dogs has been found to be low [38–41], the potential severity of antimicrobial resistant populations and high risk of direct/indirect transfer means companion animals should not be overlooked as important sources of *Salmonella*.

The isolation of *S*. Enteriditis in samples other than humans was unexpected because this serotype is believed to be exotic to Australia [8, 9], and human cases are usually attributed to travel [18]. Nevertheless, we show *S*. Enteriditis to be occurring in a number of domestic samples (food, companion animals, and wildlife). Further investigations utilising whole genome sequencing would enable a better understanding of the relevance of these sources to human cases.

This study found that a number of common serotypes isolated from humans are present in wildlife and natural environments. The low diversity of serotypes in wild mammals and the natural environment (*D* = 0.62 and 0.3, respectively) is consistent with a study that traced an outbreak of *S*. Paratyphi B Java in humans to contaminated playground sand and bandicoots in NSW [11]. The isolation of *S*. Wangata in wildlife, particularly birds, is interesting given that human case notifications of this serotype are rising in NSW (Health Protection NSW, unpublished data). This indicates that birds might be a reservoir of *S*. Wangata, however more research is warranted to test this hypothesis. The importance of birds as reservoirs for *Salmonella* has been described following a number of outbreaks linking *Salmonella* infection in people with infection in wild bird populations [36, 42, 43]. In Tasmania there have been reports of *S.* Typhimurium DT160 in wild birds and humans, [44] however there have not yet been any molecular comparisons between samples obtained from each host. Nevertheless, the high proportion of wild bird isolates serotyped as *S*. Typhimurium in this study supports the hypothesis of potential bird to human transmission. In contrast, the absence of *S*. Typhimurium from natural environment samples is interesting given the high frequency of this serotype in the majority of other sample categories. This may be explained by the varying survival capabilities of different serotypes in the environment [45, 46] and may suggest that certain serotypes are more likely to be derived from an environmental source than others.

Pet reptiles have been shown to be an important source of human salmonellosis in the US, Canada, UK and Europe [47–50], so the low proportion of highly frequent human serotypes in reptiles was unexpected. This may be partially explained by the capability of reptiles to host a wide range of serotypes, including many reptile-associated serotypes [48]. This was illustrated in the current study by the high index of diversity for wild reptiles (*D* = 0.97). However, this does not preclude them from carrying the serotypes frequently observed in humans, which are often the serotypes associated with reptile-associated transmission [50]. Another reason for this disparity could be that in NSW there are strict regulations for reptile ownership including mandatory licensing from the state government and the requirement to purchase pet reptiles from regulated sellers. These factors might present a barrier to pet reptile ownership which would reduce the contact between pet reptiles and people and therefore the amount of reptile-associated salmonellosis.

These data are not sufficient to estimate the contribution of wildlife to human salmonellosis in NSW. Future studies would benefit from consideration of the location of sampled animals because studies have shown that wildlife from urban environments are more likely to shed *Salmonella* than wildlife located in more remote locations [51–53]. Similarly, environmental contamination on-farm also has the potential to spill-over into naïve wildlife populations [27, 54–56], so wildlife in proximity to properties may be disproportionately infected. Anthropogenic factors - such as waste disposal sites and the presence of wildlife feeding stations - impact on the diet and social interactions of wildlife, which can in turn influence the rate at which wildlife are exposed to and transmit pathogens such as *Salmonella* [52, 57]. Greater resolution of the relatedness of isolates via whole genome sequencing would also facilitate an improved understanding of *Salmonella* transmission between humans and wildlife.

### Limitations

This study has a number of limitations. Firstly, each data set included in this study consisted of data collected for different reasons, during varying time frames and within different systems (Table 1). Outbreak investigations are likely to bias the types of samples tested and the number of samples from which the outbreak serotype was isolated (e.g. *S*. Paratyphi B Java). Diagnostic data are likely to miss cases because many animals remain asymptomatic during *Salmonella* infection and companion animals, livestock, and captive wildlife that are not subjected to routine pathogen screening will therefore be underrepresented. Therefore, the lack of routine surveillance of live animals in NSW - and indeed Australia - limits our understanding of *Salmonella* in domestic species. Secondly, the lack of additional epidemiological data made interpretation of some case data difficult. When there was, for example, a spike in feline isolates with the same serotype during one year (data not shown), lack of additional information meant it was not possible to determine if this was due to an outbreak or an increase in testing. Similarly, while clinically diseased animals are not expected to enter the food chain, the lack of metadata meant this was not able to be taken into consideration. As the data reflect isolates, we cannot rule out the possibility that multiple samples were collected from the same individual or site. Finally, inconsistent classification of data (particularly sample type) within and across the data sets may have resulted in misclassification. This inconsistency might have led to duplicates being included. Alternatively, isolates might have been deemed a duplicate when they were not. Since the number excluded (*n* = 863) represents a small fraction (2%) of the total isolates described here, exclusion of these isolates is unlikely to change the conclusions.

## Conclusion

This study integrates *Salmonella* data from humans, food, livestock, companion animals and wildlife for the first time in Australia. We find that surveillance data consist overwhelmingly of human data and that other areas are lacking in either data or the appropriate level of molecular screening to enable robust source attribution. Nevertheless, we find that, while foodborne transmission was strongly supported as a major source of human salmonellosis, alternative pathways such as interactions with animals and the environment need further consideration.

## Additional files

Additional file 1: A brief description of each organisation/system that provided data for the study. (DOCX 15 kb)

Additional file 2: Details of sample types included in each sample category. (DOCX 17 kb)

Additional file 3: Top ten most frequently isolated serotypes from each sample category, including number of isolates per serotype and percentage of isolates attributable to each serotype. *S*. Monophasic – other refers to serotypes, other than *S*. Typhimurium, that are missing an H antigen and are therefore not able to be typed as a particular serotype. (DOCX 54 kb)

## Abbreviations

ARWH: Australian Registry Wildlife Health
eWHIS: Electronic Wildlife Health Information System
IDEXX: IDEXX Laboratories Pty Lt
MLVA: Multi-Locus Variable number tandem repeats Assay
NCIMS: Notifiable Conditions Incident Management System
NEPSS: National Enteric Pathogen Surveillance System
NNDSS: National Notifiable Disease Surveillance System
NSW: New South Wales
NSWFA: New South Wales Food Authority
SVDL: State Veterinary Diagnostic Laboratory
