# Supplementary material for: Diversity of Salmonella serotypes from humans, food, domestic animals and wildlife in New South Wales, Australia
Source: BMC Infect Dis. 2018 Dec 5;18:623. doi: 10.1186/s12879-018-3563-1 (PMC6280480; doi:10.1186/s12879-018-3563-1)
Supplement: Supplementary file 1 — A brief description of each organisation/system that provided data for the study. (DOCX 15 kb) [file 12879_2018_3563_MOESM1_ESM.docx]

| **Data Source** | **Acronym** | **Organisation type** | **Details** | **Website** |
| --- | --- | --- | --- | --- |
| Notifiable Conditions Incident Management System, Population Health NSW, NSW Health | NCIMS | State Government | Provides the system where by all data from notifiable conditions in humans in NSW are captured. Data is provided by pathology laboratories, general practitioners and hospitals. | <http://www.health.nsw.gov.au/epidemiology/Pages/notifiable-conditions.aspx> |
| New South Wales Food Authority, Department of Primary Industries | NSWFA | State Government | Regulatory agency responsible for regulating and monitoring food safety from farm to retail. Involved in incident response and providing education and information on best food safety practices to consumers and industry. | <http://www.foodauthority.nsw.gov.au/> |
| State Veterinary Diagnostic Laboratory, Department of Primary Industries | SVDL | State Government | State run laboratory providing diagnostic testing for all animals in New South Wales. Primarily services livestock. | <http://www.dpi.nsw.gov.au/about-us/services/laboratory-services/veterinary> |
| Australian Registry Wildlife Health, Taronga Conservation Society, NSW Office of Environment and Heritage | ARWH | Conservation Society under the State office of Environment and Heritage, NSW | Conservations society that provides diagnostic pathology for wildlife, generates information and advice on key wildlife diseases, maintains archives of wildlife disease information and provides educational services. | <http://arwh.org/> |
| Electronic Wildlife Health Information System, Wildlife Health Australia | eWHIS | Not-for-profit organisation (Core funding provided mainly by Australian Government Department of Agriculture and Water Resources) | Database collating information of disease in wildlife in Australia. | <https://wildlifehealthaustralia.com.au/AboutUs.aspx> |
| IDEXX Laboratories Pty Ltd | IDEXX | Private company | Private laboratory that provides diagnostic services for veterinary clinics. | <http://www.idexx.com.au/corporate/about-idexx.html> |
| National Enteric Pathogen Surveillance System, Microbiological Diagnostic Unit Public Health Laboratory | NEPSS | Public Health Laboratory/ Database | Database for enteric diseases in human and non-human samples. Submission of samples to NEPSS is voluntary for all states other than Victoria and Tasmania. | <http://www.health.gov.au/internet/main/publishing.nsf/content/cda-cdi3001m.htm> |
