# Supplementary material for: Diversity of Salmonella serotypes from humans, food, domestic animals and wildlife in New South Wales, Australia
Source: BMC Infect Dis. 2018 Dec 5;18:623. doi: 10.1186/s12879-018-3563-1 (PMC6280480; doi:10.1186/s12879-018-3563-1)
Supplement: Supplementary file 2 — Details of sample types included in each sample category. (DOCX 17 kb) [file 12879_2018_3563_MOESM2_ESM.docx]

|  | **Sample Category** | **Sub-categories** | |
| --- | --- | --- | --- |
| 1 | Humans | Human |  |
| 2 | Animal Feed | Animal feed NFS*  Chicken feed  Cotton seed | Feather/meat meal  Meal NFS*  Water |
| 3 | Non-Animal food | Canola  Grain  Herb  Nut & Seed  Nut & Seed product  Soybean  Spice  Sunflower  Vegetable NFS*  Apple strudel  Beetroot  Carrot | Chocolate pastry  Coriander  Cucumber  Hummus  Lettuce  Mung beans  Onion  Peanuts  Capsicum  Tomato  Tabouli  Sprouts |
| 4 | Beef | Beef  Beef sausage  Beef steak | Silverside  Roast beef |
| 5 | Dairy | Cheese  Cream | Dairy NFS* |
| 6 | Poultry/eggs | Turkey mince  Duck carcass  Eggs  Egg butter  Egg shell rinse  Chicken breast fillet  Chicken drumstick  Chicken kebab  Chicken liver pate  Chicken mince | Chicken sausage  Chicken tenderloin  Chicken thigh  Chicken wing  Deep-fried ice-cream batter (made using raw egg)  Chicken carcass  Mayonnaise  Chicken product NFS* |
| 7 | Lamb/Goat | Goat  Lamb kidney | Lamb mince  Lamb ragu |
| 8 | Pork | Ham  Pork meat  Pork heart  Port kidney | Pork liver  Pork sausage  Pork intestine  Pork stomach |
| 9 | Game | Kangaroo  Crocodile | Boar |
| 10 | Seafood | Prawn  Tuna | Seafood NFS* |
| 11 | Meat unknown | Carcass  Pate  Tenderloins | Meat NFS*  Red rectangular cut meat  White rectangular cut meat |
| 12 | Mixed | Bacon and egg roll | Pork and chicken roll |
| 13 | Retail environment | Kitchen equipment  Restaurant restroom | Restaurant floor  Display bird pens |
| 14 | Farm environment | Animal habitat  Farm environment  Boot swabs | Farm equipment  Rat faeces on farm |
| 15 | Natural environment | Sand and bark | Natural water source |
| 16 | Other environment | Water NFS*  Cleaning equipment NFS* | Effluent NFS*  Environmental sample NFS* |
| 17 | Cattle | Cattle |  |
| 18 | Pig | Pigs |  |
| 19 | Poultry | Chickens broilers | Chicken layers |
| 20 | Small ruminants | Sheep  Alpaca | Goat |
| 21 | Horse | Horse |  |
| 22 | Cat | Cat |  |
| 23 | Dog | Dog |  |
| 24 | Captive Bird | Accipitriformes  Anseriformes  Caprimulgiformes  Casuariiformes  Cathartiformes  Charadriiformes  Ciconiiformes  Columbiformes | Cuculiformes  Galliformes  Gruiformes  Passeriformes  Pelecaniformes  Psittaciformes  Sphenisciformes |
| 25 | Captive Reptiles | Squamata | Testudines |
| 26 | Captive Mammals | Artiodactyla  Carnivora  Diprotodontia  Lagomorphia  Monotremata | Peramelemorphia  Perissodactyla  Primates  Rodentia  Strigiformes |
| 27 | Wild Bird | Anseriformes  Caprimulgiformes  Charadriiformes  Ciconiiformes  Columbiformes  Cuculiformes  Galliformes | Gruiformes  Passeriformes  Pelecaniformes  Psittaciformes  Sphenisciformes  Strigiformes |
| 28 | Wild Mammals | Diprotodontia  Lagomorphia  Monotremata | Peramelemorphia  Rodentia |
| 29 | Wild Reptiles | Squamata | Testudines |

*NFS = not further specified
