# Supplementary material for: Diversity of Salmonella serotypes from humans, food, domestic animals and wildlife in New South Wales, Australia
Source: BMC Infect Dis. 2018 Dec 5;18:623. doi: 10.1186/s12879-018-3563-1 (PMC6280480; doi:10.1186/s12879-018-3563-1)
Supplement: Supplementary file 3 — Top ten most frequently isolated serotypes from each sample category, including number of isolates per serotype and percentage of isolates attributable to each serotype. S. Monophasic – other refers to serotypes, other than S. Typhimurium, that are missing an H antigen and are therefore not able to be typed as a particular serotype. (DOCX 54 kb) [file 12879_2018_3563_MOESM3_ESM.docx]

| **1** | **Humans** | | **n** | **%** |
| --- | --- | --- | --- | --- |
|  | 1 | *S.* Typhimurium | 21291 | 56 |
|  | 2 | *S.* Enteritidis | 1644 | 4 |
|  | 3 | *S.* Virchow | 1279 | 3 |
|  | 4 | *S.* Birkenhead | 1175 | 3 |
|  | 5 | *S.* Infantis | 971 | 3 |
|  | 6 | *S.* Paratyphi B bv Java | 870 | 2 |
|  | 7 | *S.* Saintpaul | 738 | 2 |
|  | 8 | *S.* Bovismorbificans | 726 | 2 |
|  | 9 | *S.* Wangata | 575 | 2 |
|  | 10 | *S.* Stanley | 491 | 1 |
|  |  | Other (n=240) | 8346 | 22 |
|  |  | TOTAL | 38106 | 100 |
| **FOOD** | | | | |
| **2** | **Animal Feed** | | | |
|  | 1 | *S.* Orion | 213 | 15 |
|  | 2 | *S.* Senftenberg | 195 | 13 |
|  | 3 | *S.* Anatum | 145 | 10 |
|  | 4 | *S.* Tennessee | 137 | 9 |
|  | 5 | *S.* Agona | 130 | 9 |
|  | 6 | *S.* Liverpool | 100 | 7 |
|  | 7 | *S*. monophasic - other | 69 | 5 |
|  | 8 | *S.* Havana | 63 | 4 |
|  | 9 | *S.* Singapore | 58 | 4 |
|  | 10 | *S.* Infantis | 58 | 4 |
|  |  | Other (n=48) | 278 | 19 |
|  |  | TOTAL | 1446 | 100 |
| **3** | **Non-animal origin** | | | |
|  | 1 | *S.* Aberdeen | 226 | 20 |
|  | 2 | *S.* Agona | 139 | 12 |
|  | 3 | *S.* Typhimurium | 98 | 9 |
|  | 4 | *S.* Senftenberg | 55 | 5 |
|  | 5 | *S.* Mbandaka | 54 | 5 |
|  | 6 | *S*. monophasic - other | 52 | 5 |
|  | 7 | *S.* Havana | 43 | 4 |
|  | 8 | *S.* Hvittingfoss | 41 | 4 |
|  | 9 | *S.* Birkenhead | 32 | 3 |
|  | 10 | *S.* Zanzibar | 32 | 3 |
|  |  | Other (n=59) | 367 | 32 |
|  |  | TOTAL | 1139 | 100 |

| **4** | **Beef** | | | |
| --- | --- | --- | --- | --- |
|  | 1 | *S.* Typhimurium | 8 | 13 |
|  | 2 | *S.* Bovismorbificans | 8 | 13 |
|  | 3 | *S.* Dublin | 5 | 8 |
|  | 4 | *S.* Infantis | 4 | 6 |
|  | 5 | *S.* monophasic - other | 3 | 5 |
|  | 6 | *S.* Orion | 3 | 5 |
|  | 7 | *S.* London | 3 | 5 |
|  | 8 | *S.* Give | 3 | 5 |
|  | 9 | *S.* Poona | 2 | 3 |
|  | 10 | *S.* Heidelberg | 2 | 3 |
|  |  | Other (n=19) | 22 | 35 |
|  |  | TOTAL | 63 | 100 |
| **5** | **Dairy** | | | |
|  | 1 | *S.* Typhimurium | 7 | 39 |
|  | 2 | *S.* Uganda | 1 | 6 |
|  | 3 | *S.* Singapore | 1 | 6 |
|  | 4 | *S.* Zanzibar | 1 | 6 |
|  | 5 | *S.* Bredeney | 1 | 6 |
|  | 6 | *S.* Bovismorbificans | 1 | 6 |
|  | 7 | *S.* Tennessee | 1 | 6 |
|  | 8 | *S.* Kottbus | 1 | 6 |
|  | 9 | *S.* Warragul | 1 | 6 |
|  | 10 | *S.* Mbandaka | 1 | 6 |
|  |  | Other (n=2) | 2 | 11 |
|  |  | TOTAL | 18 | 100 |
| **6** | **Poultry/eggs** | | | |
|  | 1 | *S.* Subsp II ser 1,4,12,27:b:[e,n,x] (Sofia) | 564 | 41 |
|  | 2 | *S.* Typhimurium | 251 | 18 |
|  | 3 | *S.* Infantis | 105 | 8 |
|  | 4 | *S.* Abortusovis | 104 | 8 |
|  | 5 | *S.* monophasic - other | 70 | 5 |
|  | 6 | *S.* Montevideo | 53 | 4 |
|  | 7 | *S.* Kiambu | 30 | 2 |
|  | 8 | *S.* Anatum | 19 | 1 |
|  | 9 | *S.* Virchow | 17 | 1 |
|  | 10 | *S.* Agona | 17 | 1 |
|  |  | Other (n=38) | 140 | 10 |
|  |  | TOTAL | 1370 | 100 |

| **7** | **Lamb/goat** | | | |
| --- | --- | --- | --- | --- |
|  | 1 | *S.* Typhimurium | 7 | 21 |
|  | 2 | *S.* Reading | 3 | 9 |
|  | 3 | *S.* Bredeney | 3 | 9 |
|  | 4 | *S.* Subsp II ser 1,4,12,27:b:[e,n,x] (Sofia) | 2 | 6 |
|  | 5 | *S.* Saintpaul | 2 | 6 |
|  | 6 | *S.* Tennessee | 2 | 6 |
|  | 7 | *S.* Bovismorbificans | 1 | 3 |
|  | 8 | *S.* Give | 1 | 3 |
|  | 9 | *S.* Chester | 1 | 3 |
|  | 10 | *S.* Havana | 1 | 3 |
|  |  | Other (n=10) | 10 | 30 |
|  |  | TOTAL | 33 | 100 |
| **8** | **Pork** | | | |
|  | 1 | *S.* monophasic - other | 56 | 16 |
|  | 2 | *S.* Typhimurium | 49 | 14 |
|  | 3 | *S.* Anatum | 38 | 11 |
|  | 4 | *S.* Rissen | 36 | 10 |
|  | 5 | *S.* Infantis | 32 | 9 |
|  | 6 | *S.* London | 28 | 8 |
|  | 7 | *S.* Derby | 19 | 5 |
|  | 8 | *S.* Johannesburg | 14 | 4 |
|  | 9 | *S.* Stanley | 13 | 4 |
|  | 10 | *S.* Ohio | 8 | 2 |
|  |  | Other (n=22) | 55 | 16 |
|  |  | TOTAL | 348 | 100 |
| **9** | **Game meat** | | | |
|  | 1 | *S.* Urbana | 3 | 15 |
|  | 2 | *S.* Zanzibar | 1 | 5 |
|  | 3 | *S.* Saintpaul | 1 | 5 |
|  | 4 | *S.* Onderstepoort | 1 | 5 |
|  | 5 | *S.* Bovismorbificans | 1 | 5 |
|  | 6 | *S.* Subsp IIIb ser 61:k:1,5,7 | 1 | 5 |
|  | 7 | *S.* Bredeney | 1 | 5 |
|  | 8 | *S.* Muenchen | 1 | 5 |
|  | 9 | *S.* Chester | 1 | 5 |
|  | 10 | *S.* Rubislaw | 1 | 5 |
|  |  | Other (n=8) | 8 | 40 |
|  |  | TOTAL | 20 | 100 |

| **10** | **Seafood** | | | |
| --- | --- | --- | --- | --- |
|  | 1 | *S.* Paratyphi B bv Java | 12 | 44 |
|  | 2 | *S.* Agona | 7 | 26 |
|  | 3 | *S.* Typhimurium | 2 | 7 |
|  | 4 | *S.* Virchow | 2 | 7 |
|  | 5 | *S.* Chailey | 1 | 4 |
|  | 6 | *S.* Weltevreden | 1 | 4 |
|  | 7 | *S.* Bareilly | 1 | 4 |
|  | 8 | *S.* Subsp II ser 17:g,t:[e,n,x,z15] | 1 | 4 |
|  | 9 |  |  |  |
|  | 10 |  |  |  |
|  |  |  |  |  |
|  |  | TOTAL | 27 | 100 |
| **11** | **Meat (unknown)** | | | |
|  | 1 | *S.* Subsp II ser 1,4,12,27:b:[e,n,x] (Sofia) | 42 | 27 |
|  | 2 | *S.* London | 25 | 16 |
|  | 3 | *S.* Typhimurium | 22 | 14 |
|  | 4 | *S.* Infantis | 10 | 7 |
|  | 5 | *S.* Abortusovis | 7 | 5 |
|  | 6 | *S.* Orion | 4 | 3 |
|  | 7 | *S.* monophasic - other | 3 | 2 |
|  | 8 | *S.* Johannesburg | 3 | 2 |
|  | 9 | *S.* Havana | 3 | 2 |
|  | 10 | *S.* Anatum | 3 | 2 |
|  |  | Other (n=22) | 31 | 20 |
|  |  | TOTAL | 153 | 100 |
| **12** | **Mixed** | | | |
|  | 1 | *S.* Typhimurium | 25 | 58 |
|  | 2 | *S.* Infantis | 3 | 7 |
|  | 3 | *S.* Virchow | 2 | 5 |
|  | 4 | *S.* Anatum | 2 | 5 |
|  | 5 | *S.* Montevideo | 2 | 5 |
|  | 6 | *S.* Newport | 2 | 5 |
|  | 7 | *S.* Subsp II ser 1,4,12,27:b:[e,n,x] (Sofia) | 1 | 2 |
|  | 8 | *S.* Senftenberg | 1 | 2 |
|  | 9 | *S.* Give | 1 | 2 |
|  | 10 | *S.* Enteritidis | 1 | 2 |
|  |  | Other (n=3) | 3 | 7 |
|  |  | TOTAL | 43 | 100 |

| **ENVIRONMENT** | | | | |
| --- | --- | --- | --- | --- |
| **13** | **Retail** | | | |
|  | 1 | *S.* Typhimurium | 98 | 52 |
|  | 2 | *S.* Infantis | 48 | 26 |
|  | 3 | *S.* Virchow | 8 | 4 |
|  | 4 | *S.* Bovismorbificans | 7 | 4 |
|  | 5 | *S.* Bareilly | 6 | 3 |
|  | 6 | *S.* Hvittingfoss | 5 | 3 |
|  | 7 | *S.* Subsp II ser 1,4,12,27:b:[e,n,x] (Sofia) | 2 | 1 |
|  | 8 | *S.* Singapore | 2 | 1 |
|  | 9 | *S.* Kiambu | 2 | 1 |
|  | 10 | *S.* Mbandaka | 2 | 1 |
|  |  | Other (n=8) | 8 | 4 |
|  |  | TOTAL | 188 | 100 |
| **14** | **Farm** | | | |
|  | 1 | *S.* Infantis | 649 | 20 |
|  | 2 | *S.* Typhimurium | 645 | 20 |
|  | 3 | *S.* monophasic - other | 361 | 11 |
|  | 4 | *S.* Agona | 162 | 5 |
|  | 5 | *S.* Kiambu | 159 | 5 |
|  | 6 | *S.* Ohio | 154 | 5 |
|  | 7 | *S.* Mbandaka | 141 | 4 |
|  | 8 | *S.* Singapore | 136 | 4 |
|  | 9 | *S.* Virchow | 84 | 3 |
|  | 10 | *S.* Senftenberg | 80 | 2 |
|  |  | Other (n=58) | 640 | 20 |
|  |  | TOTAL | 3211 | 100 |
| **15** | **Natural** | | | |
|  | 1 | *S.* Paratyphi B bv Java | 93 | 84 |
|  | 2 | *S.* monophasic - other | 7 | 6 |
|  | 3 | *S.* Bovismorbificans | 2 | 2 |
|  | 4 | *S.* Victoria | 1 | 1 |
|  | 5 | *S.* Subsp II ser 21:z10:z6 | 1 | 1 |
|  | 6 | *S.* monophasic - Typhimurium | 1 | 1 |
|  | 7 | *S.* Reading | 1 | 1 |
|  | 8 | *S.* Subsp II ser 42:g,t:- | 1 | 1 |
|  | 9 | *S.* Welikade | 1 | 1 |
|  | 10 | *S.* Give | 1 | 1 |
|  |  | Other (n=2) | 2 | 2 |
|  |  | TOTAL | 111 | 100 |

| **16** | **Other** | | | |
| --- | --- | --- | --- | --- |
|  | 1 | *S.* monophasic - other | 230 | 27 |
|  | 2 | *S.* Singapore | 168 | 20 |
|  | 3 | *S.* Livingstone | 127 | 15 |
|  | 4 | *S.* Typhimurium | 65 | 8 |
|  | 5 | *S.* Worthington | 29 | 3 |
|  | 6 | *S.* Agona | 26 | 3 |
|  | 7 | *S.* Mbandaka | 25 | 3 |
|  | 8 | *S.* Tennessee | 19 | 2 |
|  | 9 | *S.* Infantis | 16 | 2 |
|  | 10 | *S.* Kentucky | 15 | 2 |
|  |  | Other (n=37) | 130 | 15 |
|  |  | TOTAL | 850 | 100 |
| **ANIMAL** | | | | |
| **17** | **Cattle** | | | |
|  | 1 | *S.* Typhimurium | 191 | 32 |
|  | 2 | *S.* Dublin | 152 | 26 |
|  | 3 | *S.* Bovismorbificans | 73 | 12 |
|  | 4 | *S.* Anatum | 18 | 3 |
|  | 5 | *S.* Give | 16 | 3 |
|  | 6 | *S.* Infantis | 14 | 2 |
|  | 7 | *S.* Newport | 13 | 2 |
|  | 8 | *S.* Orion | 12 | 2 |
|  | 9 | *S.* Mbandaka | 10 | 2 |
|  | 10 | *S.* Zanzibar | 10 | 2 |
|  |  | Other (n=28) | 87 | 15 |
|  |  | TOTAL | 596 | 100 |
| **18** | **Pig** | | | |
|  | 1 | *S.* monophasic - Typhimurium | 169 | 24 |
|  | 2 | *S.* Rissen | 119 | 17 |
|  | 3 | *S.* Typhimurium | 67 | 10 |
|  | 4 | *S.* Derby | 40 | 6 |
|  | 5 | *S.* Bredeney | 38 | 5 |
|  | 6 | *S.* Johannesburg | 31 | 4 |
|  | 7 | *S.* Bovismorbificans | 29 | 4 |
|  | 8 | *S.* Ohio | 28 | 4 |
|  | 9 | *S.* Anatum | 28 | 4 |
|  | 10 | *S.* Infantis | 26 | 4 |
|  |  | Other (n=20) | 128 | 18 |
|  |  | TOTAL | 703 | 100 |

| **19** | **Poultry** | | | |
| --- | --- | --- | --- | --- |
|  | 1 | *S.* Typhimurium | 33 | 61 |
|  | 2 | *S.* Give | 5 | 9 |
|  | 3 | *S.* Orion | 4 | 7 |
|  | 4 | *S.* Subsp II ser 1,4,12,27:b:[e,n,x] (Sofia) | 2 | 4 |
|  | 5 | *S.* Kiambu | 2 | 4 |
|  | 6 | *S.* Mbandaka | 2 | 4 |
|  | 7 | *S.* Senftenberg | 1 | 2 |
|  | 8 | *S.* Agona | 1 | 2 |
|  | 9 | *S.* Bovismorbificans | 1 | 2 |
|  | 10 | *S.* Infantis | 1 | 2 |
|  |  | Other (n=2) | 2 | 4 |
|  |  | TOTAL | 54 | 100 |
| **20** | **Small ruminants** | | | |
|  | 1 | *S.* Typhimurium | 73 | 49 |
|  | 2 | *S.* Bovismorbificans | 37 | 25 |
|  | 3 | *S.* Infantis | 17 | 11 |
|  | 4 | *S.* Tennessee | 4 | 3 |
|  | 5 | *S.* Muenchen | 2 | 1 |
|  | 6 | *S.* Dublin | 2 | 1 |
|  | 7 | *S.* Orientalis | 2 | 1 |
|  | 8 | *S.* Zanzibar | 1 | 1 |
|  | 9 | *S.* Chester | 1 | 1 |
|  | 10 | *S.* Amsterdam | 1 | 1 |
|  |  | Other (n=9) | 9 | 6 |
|  |  | TOTAL | 149 | 100 |
| **21** | **Horse** | | | |
|  | 1 | *S.* Typhimurium | 18 | 32 |
|  | 2 | *S.* Bovismorbificans | 5 | 9 |
|  | 3 | *S.* Anatum | 5 | 9 |
|  | 4 | *S.* Orion | 4 | 7 |
|  | 5 | *S.* Infantis | 3 | 5 |
|  | 6 | *S.* Montevideo | 2 | 4 |
|  | 7 | *S.* Muenchen | 2 | 4 |
|  | 8 | *S.* Muenster | 2 | 4 |
|  | 9 | *S.* Give | 2 | 4 |
|  | 10 | *S.* Agona | 2 | 4 |
|  |  | Other (n=9) | 12 | 21 |
|  |  | TOTAL | 57 | 100 |

| **22** | **Cat** | | | |
| --- | --- | --- | --- | --- |
|  | 1 | *S.* Typhimurium | 78 | 52 |
|  | 2 | *S.* Chester | 12 | 8 |
|  | 3 | *S.* Virchow | 7 | 5 |
|  | 4 | *S.* Infantis | 6 | 4 |
|  | 5 | *S.* monophasic - other | 5 | 3 |
|  | 6 | *S.* Bovismorbificans | 4 | 3 |
|  | 7 | *S.* monophasic - Typhimurium | 4 | 3 |
|  | 8 | *S.* Muenchen | 4 | 3 |
|  | 9 | *S.* Subsp II ser 1,4,12,27:b:[e,n,x] (Sofia) | 3 | 2 |
|  | 10 | *S.* Waycross | 3 | 2 |
|  |  | Other serotype (n=20) | 24 | 16 |
|  |  | TOTAL | 150 | 100 |
| **23** | **Dog** | | | |
|  | 1 | *S.* Typhimurium | 71 | 19 |
|  | 2 | *S.* Bovismorbificans | 23 | 6 |
|  | 3 | *S.* Virchow | 20 | 5 |
|  | 4 | *S.* Infantis | 19 | 5 |
|  | 5 | *S.* Subsp II ser 4,12:b:- | 18 | 5 |
|  | 6 | *S.* Subsp II ser 1,4,12,27:b:[e,n,x] (Sofia) | 13 | 3 |
|  | 7 | *S.* Aberdeen | 12 | 3 |
|  | 8 | *S.* Agona | 11 | 3 |
|  | 9 | *S.* Saintpaul | 10 | 3 |
|  | 10 | *S.* Dublin | 10 | 3 |
|  |  | Other (n=56) | 170 | 45 |
|  |  | TOTAL | 377 | 100 |
| **24** | **Captive bird** | | | |
|  | 1 | *S.* Typhimurium | 63 | 66 |
|  | 2 | *S.* Wangata | 9 | 9 |
|  | 3 | *S.* Bovismorbificans | 2 | 2 |
|  | 4 | *S.* Senftenberg | 2 | 2 |
|  | 5 | *S.* Kottbus | 2 | 2 |
|  | 6 | *S.* Infantis | 2 | 2 |
|  | 7 | *S.* Mbandaka | 2 | 2 |
|  | 8 | *S.* Subsp IV ser 1,44:z4,z32:- | 1 | 1 |
|  | 9 | *S.* Virchow | 1 | 1 |
|  | 10 | *S.* Subsp IIIb ser 61:z52:z53 | 1 | 1 |
|  |  | Other (n=11) | 11 | 11 |
|  |  | TOTAL | 96 | 100 |

| **25** | **Captive reptile** | | | |
| --- | --- | --- | --- | --- |
|  | 1 | *S.* Subsp IIIa ser 41:z4,z23:- | 24 | 10 |
|  | 2 | *S.* Subsp IV ser 43:z4,z23:- | 19 | 8 |
|  | 3 | *S.* Subsp IIIb ser 18:l,v:z | 9 | 4 |
|  | 4 | *S.* Subsp IIIb ser 48:z52:z | 8 | 3 |
|  | 5 | *S.* Adelaide | 8 | 3 |
|  | 6 | *S.* Subsp IIIb ser 35:k:z53 | 7 | 3 |
|  | 7 | *S.* Subsp IIIb ser 61:z52:z53 | 7 | 3 |
|  | 8 | *S.* Havana | 6 | 3 |
|  | 9 | *S.* Subsp IIIb ser 61:r:z53 | 6 | 3 |
|  | 10 | *S.* Muenchen | 6 | 3 |
|  |  | Other (n=78) | 136 | 58 |
|  |  | TOTAL | 236 | 100 |
| **26** | **Captive mammal** | | | |
|  | 1 | *S.* Typhimurium | 16 | 18 |
|  | 2 | *S.* Give | 9 | 10 |
|  | 3 | *S.* Saintpaul | 7 | 8 |
|  | 4 | *S.* Bovismorbificans | 7 | 8 |
|  | 5 | *S.* Agona | 5 | 6 |
|  | 6 | *S.* Havana | 5 | 6 |
|  | 7 | *S.* Hvittingfoss | 3 | 3 |
|  | 8 | *S.* Kiambu | 3 | 3 |
|  | 9 | *S.* Orion | 3 | 3 |
|  | 10 | *S.* Adelaide | 2 | 2 |
|  |  | Other (n=22) | 28 | 32 |
|  |  | TOTAL | 88 | 100 |
| **27** | **Wild bird** | | | |
|  | 1 | *S.* Typhimurium | 40 | 53 |
|  | 2 | *S.* monophasic - other | 6 | 8 |
|  | 3 | *S.* Paratyphi B bv Java | 5 | 7 |
|  | 4 | *S.* monophasic - Typhimurium | 3 | 4 |
|  | 5 | *S.* Infantis | 3 | 4 |
|  | 6 | *S.* Chester | 2 | 3 |
|  | 7 | *S.* Orion | 2 | 3 |
|  | 8 | *S.* Adelaide | 2 | 3 |
|  | 9 | *S.* Singapore | 2 | 3 |
|  | 10 | *S.* Newport | 1 | 1 |
|  |  | Other (n=10) | 12 | 16 |
|  |  | TOTAL | 76 | 100 |

| **28** | **Wild reptile** | | | |
| --- | --- | --- | --- | --- |
|  | 1 | *S.* monophasic - other | 29 | 45 |
|  | 2 | *S.* Adelaide | 8 | 12 |
|  | 3 | *S.* Subsp IV ser 43:z4,z23:- | 3 | 5 |
|  | 4 | *S.* Paratyphi B bv Java | 3 | 5 |
|  | 5 | *S.* Montevideo | 2 | 3 |
|  | 6 | *S.* Subsp IV ser rough:z4,z23:- | 2 | 3 |
|  | 7 | *S.* monophasic - Typhimurium | 2 | 3 |
|  | 8 | *S.* Dublin | 2 | 3 |
|  | 9 | *S.* Havana | 2 | 3 |
|  | 10 | *S.* Rubislaw | 2 | 3 |
|  |  | Other (n=10) | 10 | 15 |
|  |  | TOTAL | 65 | 100 |
| **29** | **Wild mammal** | | | |
|  | 1 | *S.* Paratyphi B bv Java | 60 | 61 |
|  | 2 | *S.* Sylvania | 7 | 7 |
|  | 3 | *S.* Bovismorbificans | 6 | 6 |
|  | 4 | *S.* Typhimurium | 5 | 5 |
|  | 5 | *S.* Dublin | 4 | 4 |
|  | 6 | *S.* Potsdam | 2 | 2 |
|  | 7 | *S.* Orion | 2 | 2 |
|  | 8 | *S.* Zanzibar | 2 | 2 |
|  | 9 | *S.* Muenchen | 2 | 2 |
|  | 10 | *S.* Give | 1 | 1 |
|  |  | Other (n=8) | 8 | 8 |
|  |  | TOTAL | 99 | 100 |
